# Development and evaluation of an interdisciplinary teaching model via 3D printing

**DOI:** 10.1002/cre2.334

**Published:** 2020-10-27

**Authors:** Marcel Reymus, Anja Liebermann, Christian Diegritz, Andreas Keßler

**Affiliations:** ^1^ Department of Conservative Dentistry and Periodontology University Hospital, Ludwig‐Maximilians‐University Munich Germany; ^2^ Department of Prosthetic Dentistry University Hospital, Ludwig‐Maximilians‐University Munich Germany

**Keywords:** 3D printing, CAD/CAM, dental education, DICOM, interdisciplinary, STL

## Abstract

The investigation aimed to assess the feasibility of creating an interdisciplinary training model simulating endodontic, restorative as well as implantologic treatment procedures by using 3D printing technology. A CBCT scan of the mandible of a real patient was initially taken. The generated DICOM‐data were converted to a STL‐file, which was further processed to design spaces for exchangeable replica teeth, a bone segment and an adapter to fix the model in a mannequin's head. After the manufacturing process, the model was evaluated by dental students performing a root canal treatment, the insertion of a glass fibre post and the insertion of an implant. The workflow allowed a simple and cost‐effective way of manufacturing a single model, which is suitable for several training scenarios in the fields of endodontics, prosthodontics and implantology. The model was rated as being comparable to the real patient situation and offers repetitive treatment simulations. The present workflow is a feasible way of using DICOM‐data and 3D printing for an interdisciplinary training model. The dental schools can design models according to their own curriculum and put the focus on a patient centered education.

## 1 INTRODUCTION

For their pre‐clinical curriculum, dental schools are in need of models that simulate patients' care and a variety of pathologies in a realistic manne (Yang et al., 2018). The aim of these simulations is to enable dental students a smoother transition into the clinical setting, to broaden their experience by offering repetitive training which imitates selected treatment steps and finally to give them some confidence before their first dental care on a real patient.

Most dental schools train their under‐graduate students on so called typodonts provided by manufacturers like Frasaco (Tettnang, Germany) and KaVo (Biberbach, Germany). These models are suitable for a wide range of restorative and prosthetic exercises and permit repetitive training since the replica teeth are exchangeable by screws. They are, however, not realistic down to the last detail as they present a perfectly shaped orthognathic denture without any deformities. Furthermore, endodontic treatment procedures cannot be simulated satisfactorily with such models as the roots are shortened in length with missing root canals. Hence, the most realistic scenario still remains the training with extracted human teeth—a method which for decades has been the gold standard for the pre‐clinical curriculum since it creates a perfect setting to train reading radiographs, caries removal, filling therapy, and root canal treatment (Wolgin, Wiedemann, Frank, Wrbas, & Kielbassa, 2015). Nevertheless, in recent years the disadvantages of extracted human teeth have become more noticeable. The selection of suitable teeth for training purposes is time‐consuming, their origin in high quantities poses ethical questions and the potential of cross‐infections is a danger to unexperienced undergraduate students (DeWald, 1997). Consequently, alternatives to extracted human teeth have been evaluated over the last years. For endodontic training commercial replicas have been introduced into the pre‐clinical setting. They are said to be comparable to human teeth regarding the outcome in training (Anderson, Wealleans, & Ray, 2018; Bitter, Gruner, Wolf, & Schwendicke, 2016; Tchorz, Brandl, Ganter, et al., 2015). One possible solution that enables dental educational institutions to offer their students highly cost‐effective training measures lies in the in‐house production of replica teeth using 3D printing technology (Reymus et al., 2019). In this line, Lambrecht et al. have presented a surgical model based on a Cone‐Beam Computed Tomography (CBCT) of a real patient (Lambrecht, Berndt, Christensen, & Zehnder, 2010). Werz et al. printed a 3D simulator for surgeons in order to simulate sinus lift or third molar extraction (Werz, Zeichner, Berg, Zeilhofer, & Thieringer, 2018). Kröger et al. designed an interdisciplinary model for training caries and crown removal as well as veneer preparation based on the surface scan of a patient (Kröger, Dekiff, & Dirksen, 2017).

Since the call of action of the ADEE (Association of Dental Education in Europe) to integrate implant dentistry into the university curricula, different simulations have been presented (Mattheos, De Bruyn, Hultin, et al., 2014). While one model offers training in a mannequin's head (Güth, Ponn, Mast, et al., 2010), another is a digital approach by haptic feedback (Mattheos et al., 2014). Though all of those presented models offer good simulation of specific treatment scenarios, an interdisciplinary approach is still lacking.

Therefore, the aim of this contribution was to assess the feasibility of creating a training model, which offers the possibility to simulate several treatment procedures by using a DLP (Digital Light Processing) printer and to evaluate the model by dental students.

## 2 MATERIAL AND METHODS

The study has been approved by the ethic committee of the University Hospital LMU Munich under the Project‐Nr 19–410 UE.

**FIGURE 1:**
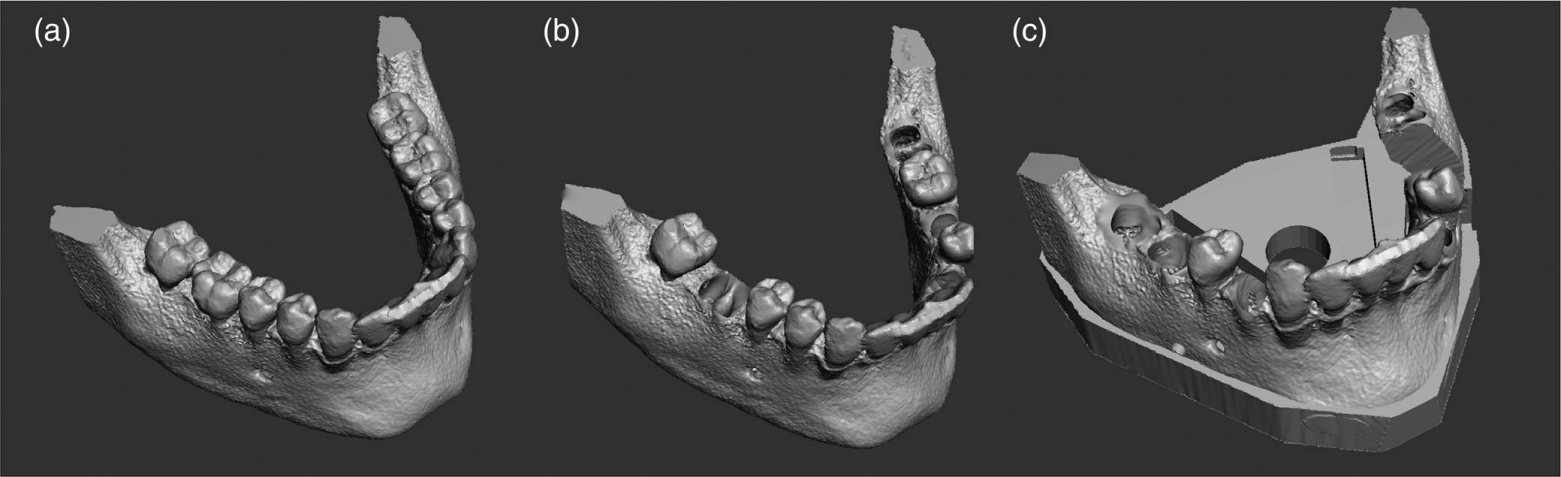
(a‐c) Workflow from the original mandible arch to the final model

A CBCT (Kodak 9,300, 10x5x5 cm, 90 kV, 3,2 mA, 8 s, 311 mGy/cm^2^, Kodak, Rochester, New York) of the mandible of one of the authors was taken after his explicit approval, facing orthodontic treatment. The generated DICOM files were imported into the open‐source software *Slicer* for Mac (www.slicer.org) which offers the possibility to convert DICOM‐data into one STL‐file (Standad Tesselation Language). Subsequently, the generated STL‐file was imported into the software *Meshmixer* for Mac 11.0 (Autodesk, San Rafael, California) (Figure 1a). Using this software several teeth (left second and first molar, left second premolar and canine, right first premolar and right first and second molar) were cut out of the STL‐mesh and the resulting holes were digitally closed.

In the next step, with the exception of the first left molar, sound extracted human teeth corresponding to the teeth mentioned above were selected. To ensure later patency of the reproduced canals, a small access cavity was prepared. The root canals were manually prepared with files up to ISO size 20, rinsed with sodium hypochlorite and dried with paper point tips. A cotton pellet of the size of the original pulp chamber was inserted into the access cavity, which was then closed with a radiopaque filling material (Tetric EvoCeram, Ivoclar‐Vivadent, Schaan, Liechtenstein). A CBCT of the extracted and prepared teeth was taken with a small field‐of‐view (Kodak 9,300, 5 × 5 × 5 cm, 78 kV, 6.3 mA, 20s) and the DICOM files were converted to STL in the same way as mentioned above. These files were appended to the STL‐file of the previously modified mandible in *Meshmixer*. The single teeth were positioned on the corresponding regions of the jaw and the function “boolean difference” was applied to cut out simulated tooth sockets corresponding in size and shape to the extracted and digitized teeth (Figure 1b).

A plate fitting the size of the mandible was designed in the software *Meshmixer* and combined with the model. The plate was subsequently prepared to leave spaces for an adapter to fix the model to a mannequin's head and for an x‐ray sensor on the right and left oral side (Figure 1c). A hole with a diameter of 2 mm through the cortical bone in the region apical to the right second molar was designed. Finally, a segment containing the tooth socket of the left first molar was cut out of the model and exported as a single STL file. The generated STL‐file was imported into the software *Meshmixer* and was further processed. In order to simulate cortical and cancellous bone structure in the bone segment, the tooth socket was removed, and the crestal area was closed in accordance with a cortical bone structure. The cancellous parts partially represented in the initial STL‐file were deleted und changed in a printable uniform honeycomb structure in order to better simulate the bone density.

The single components (i.e., the mandible model, the single teeth including (a) one for the endodontic treatment with an intact crown, (b) one supragingivally destroyed tooth for post insertion, and (c) the bone segment for implantation simulation) were imported to *Netfabb Professional* (Autodesk, San Rafael, California) and prepared for additive manufacturing.

All objects were manufactured with the 3D printer D 20 II (Rapidshape, Heimsheim, Germany). The resin *NextDent Model* (NextDent, Soesterberg, the Netherlands) was used for the reproduction of the mandible model, the resin *NextDent C&B* (NextDent) for the single replica teeth and the resin *NextDent Ortho Clear* (NextDent) for the segment. After the manufacturing process all objects were cleaned for 5 minutes in an ultra‐sonic activated ethanol 98% bath and post‐cured for 30 minutes (LC‐3D Print Box, NextDent). Subsequently, the adapter for the fixation to the mannequin's head was glued to the prefabricated space and the single replica teeth as well as the bone segment were inserted into the corresponding spaces. A wire of 2 mm in diameter was bonded into the prefabricated hole. The wire used has the appropriate geometry for connection to an electronic apex locator (EAL) (Raypex 6, VDW, Munich, Germany). When all components were combined, a gingiva mask was waxed‐up upon the model. Afterwards, the entire model was scanned (Activity 885 Mark 2, Smartoptics, Bochum, Germany) and the gingiva mask digitally extracted as one single STL‐file. This mask was then printed in the same manner as described above (D20 II, NextDent Gingiva).

The model was used for three different students' training purposes. After each training, the participants received a survey for evaluating the model (Appendix 1–3).

Forty‐eight dental students within their third year at university received extracted human teeth for simulating several endodontic treatment steps: radiographic diagnosis, opening of the pulp chamber, negotiating of the root canals, determination of working length by using an EAL, instrumentation with rotary files and obturation. Afterwards they received the 3D printed model for training the same treatment steps on the replicas. Finally, they were asked to compare their training on extracted human teeth to the one on the replicas.

Thirty‐four students within their fourth year trained the insertion of a post into a root‐canal treated supra‐gingivally destroyed tooth with missing crown as classic indication. They performed all necessary treatment steps for post insertion: X‐ray measurement of the root canal length, determination of the initial post length (4–5 mm apical seal), removal of the gutta‐percha with subsequent X‐ray measurement and final determination of the post length, enlargement of the root canal to the planned ISO size 90 to working length and placement of a glass fibre post with final X‐ray control. Since they had clinical experience with this type of treatment, they were asked to evaluate the model by comparing it to a real patient's treatment.

Finally, twelve students within their fifth year used the model for an implant hands‐on course. Initially, the students received a lecture on implantology, in which the implant positioning and insertion with and without drilling templates was discussed. Afterwards the students performed a free hand and partially guided insertion of an implant (Straumann tissue level implant; 10 mm length, diameter: 4.1 mm, Straumann, Basel, Switzerland) in the model. Subsequently they were asked about the benefit of the 3D printed model for the implantation course.

## 3 RESULTS

**FIGURE 2:**
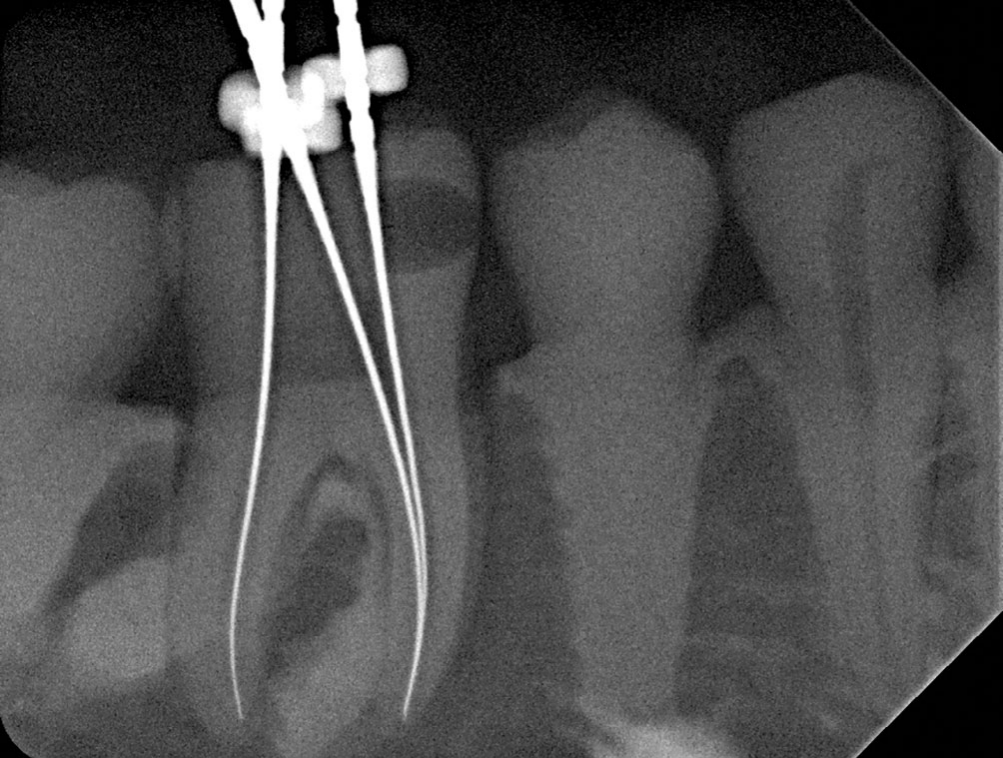
Radiopacity of the resin with a detectable carious lesion on the lower right first molar and inserted endodontic instruments

The presented workflows allowed a simple and cost‐effective way for the manufacturing of a simulation model, which was suitable for several interdisciplinary training scenarios. The resin is suitable for cutting with all common dental, endodontic or implant instruments; there is no burning or smearing effect. The radiopacity of the resin is also suitable for reading x‐rays (Figure 2). The overall raw material costs for the mandible model are about 15USD and for one replica tooth about 1USD. The material is printable on SLA and DLP devices, which are available for up to 4,000 USD (as of September 2020).

**FIGURE 3:**
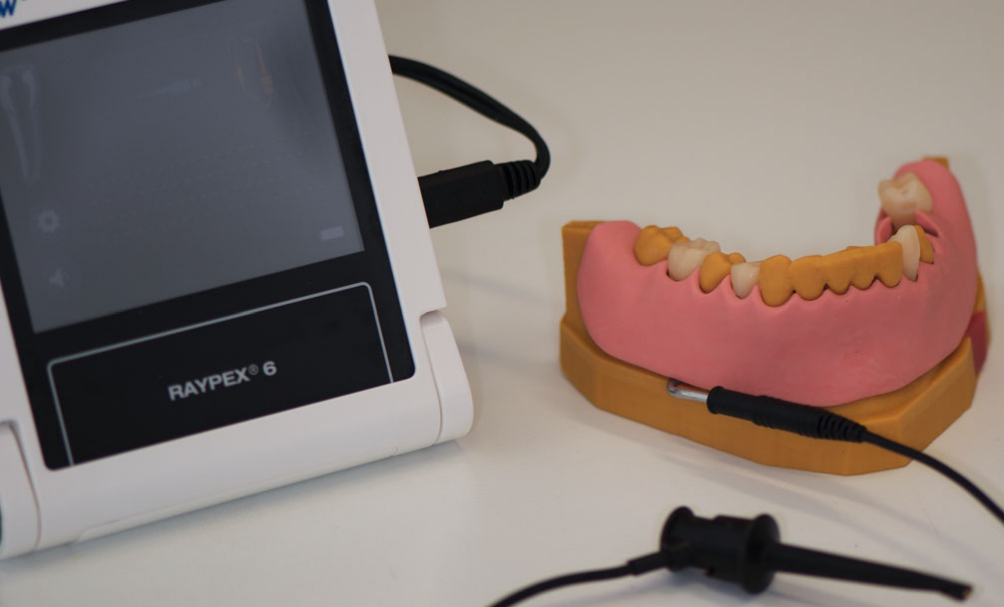
Apex locator connected to model

**FIGURE 4:**
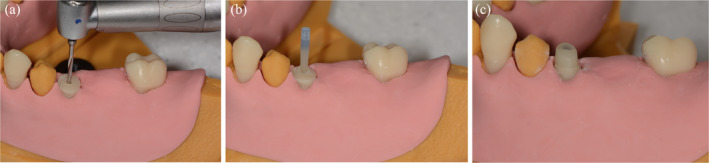
Post preparation (a), Post insertion (b), Preparation (c)

**FIGURE 5:**
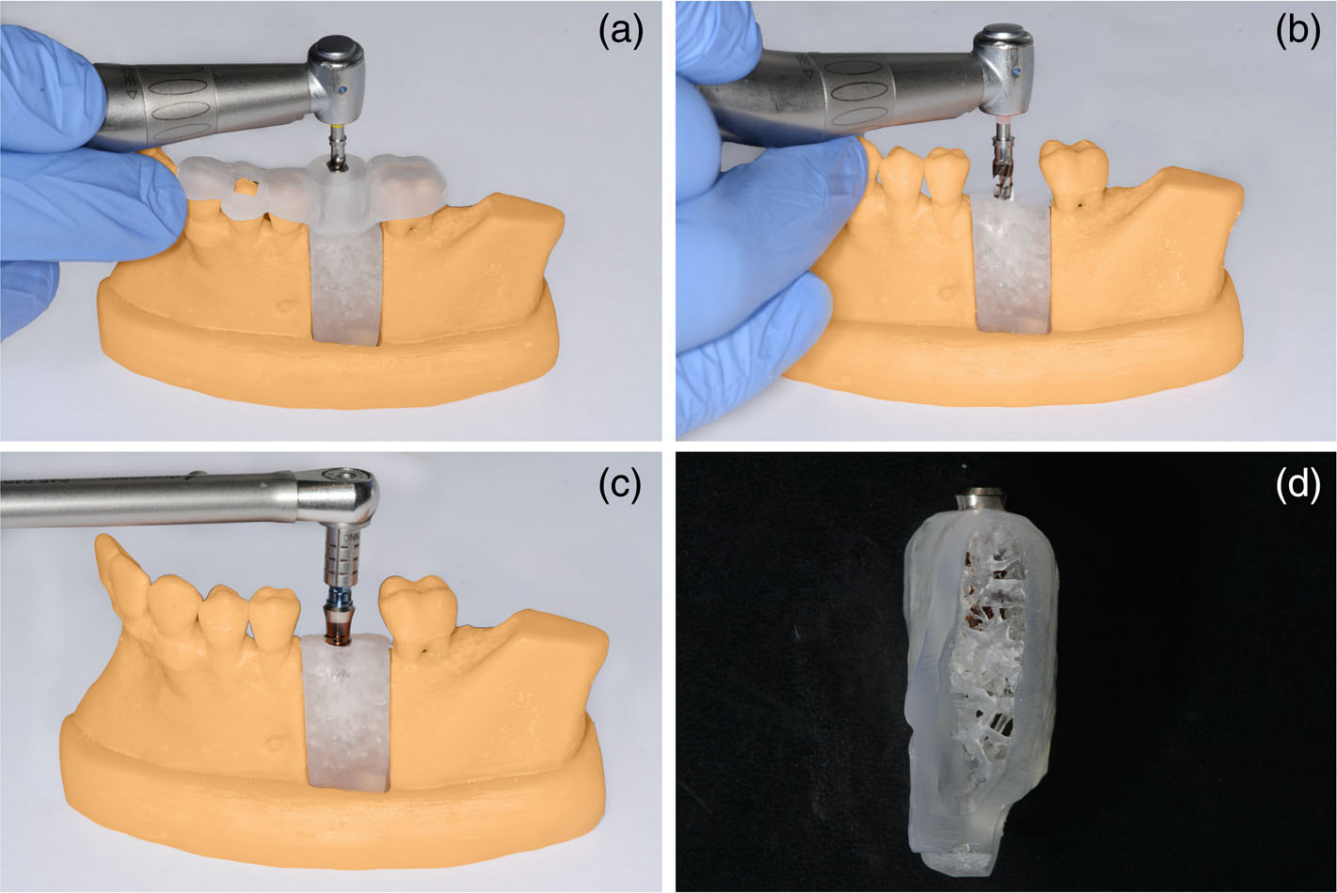
Guided pilot drill (a), implant bed preparation (b), insertion of the implant (c), sagittal view on the inserted implant with simulated corticalis and spongiosa bone structure (d)

The surveys with the corresponding results are presented in Appendix 1–3. Taking into account the evaluations of all three training procedures the model was rated to be realistic (86%). The radiographic diagnosis was equally rated to be realistic (78%). Regarding the endodontic training, the radiographic working length determination was assessed as being unrealistic (23% very similar to real teeth, 73% similar, 5% not similar). The use of the EAL worked for all students and was highly appreciated (Figure 3). Negotiation and instrumentation of the canals was feasible and rated as being similar to real teeth (33% highly realistic to real teeth, 60% realistic, 7% not realistic at all). The hardness of the replica was valued as being rather realistic (23% very similar to real teeth, 46% similar, 31% not similar). The added value of the model for endodontic training was highly appreciated (52% high added value, 45% some added value, 3% no added value). Regarding the training of post insertion, the hardness of the tooth was rated as being rather similar to real teeth (12% highly realistic to real teeth, 53% realistic, 35% not at all realistic). The drilling procedure was valued as being very realistic (44% highly realistic to real teeth, 41% realistic, 15% not realistic at all) (Figure 4). The added value of the model for training post insertion was appreciated (21% high added value, 76% some added value, 3% no added value). The majority of students (76%) felt better prepared for post insertion on a patient after the training than before.

Regarding the implantology training (Figure 5), 42% of the students saw a high value in the implant model and 58% valued an advantage in some manner. 17% agreed to the question if the design of the cortical and cancellous bone was realistic and 83% agreed partially to this question. All students attributed great added value to the guided implantology in contrast to free hand drilling. The students also stated that the individual steps of the implantation were more comprehensible to them after the exercise (75% agree, 25% partially agree, 0% not agree).

## 4 DISCUSSION

The objective of this study was to assess the feasibility of a new workflow for the production of an interdisciplinary training model by using 3D printing technology and to receive an evaluation of this model by dental students. For dental schools the creation and production of their own training models according to the specific curriculum can be highly beneficial. However, the workflow for creating such models must be feasible and the simulations must be as realistic as possible. All software applications used in this contribution were user‐friendly and available online free of charge. The 3D printer can be used for the production of educational models as well as for a range of various treatment options, like bite splints, dental casts and temporary crowns and bridges. By using exchangeable parts, solely the objects required for a specific training need to be manufactured while the mandible model itself can be reused. It can be mounted on a mannequin's head (e.g., Frasaco P‐6) making its use even more realistic. Furthermore, the model allows for the simulation of various treatment options: restorative or prosthetic procedures on the replica teeth, insertion of a dental implant in the segment part and endodontic procedures including the use of an EAL and the insertion of a post. Therefore, it offers more advantages over previously presented models (Güth et al., 2010; Kröger et al., 2017; Lambrecht et al., 2010).

The model includes several teeth for simulating endodontic treatment. The benefits to students from such printed teeth for endodontic exercises were described in a previous investigation (Reymus et al., 2019). Students especially favoured their availability and the fairness due to standardization. Since the model is based on a CBCT of the jaw of a real patient, radiographs appear realistic. Nevertheless, one point of criticism was the limited radioopacity as well as the reduced hardness of the model. Both points of concern have been stated by previous studies (Al‐Sudani & Basudan, 2017; Nassri, Carlik, da Silva, et al., 2008). One promising solution to overcome these problems has been published by Robberecht et al. (Robberecht et al., 2017). They used a ceramic material for imitating human dentin and showed comparable hardness values and a good radiopacity. One the other hand, the model allowed the connection of an EAL. This possibility was highly appreciated by the students and made the model more similar to the clinical setting of an endodontic treatment.

The training of post insertion allowed radiographic diagnosis of the existing root canal filling and planning of the final post length with apical sealing, root canal post preparation and the adhesive post insertion. For this training the replica's hardness was equally rated as being softer than human dentin. According to students' evaluation, this made the drilling procedure easier. However, if the axis of the root was not fully taken into consideration the easier drilling promoted a faster perforation of the replica. Consequently, the alleged disadvantage of a lower hardness might be even an advantage for training procedures leading to a slower and more cautious root canal post preparation clinically.

The implant drilling simulation in a bone fragment, which can be individually designed in terms of its external dimensions and division into cortical and cancellous bone, gives the clinician the opportunity to practice implant placement and subsequent bone management in clinically relevant and challenging situations. Pre‐interventional working models should put surgeons in a position to predict and secure their procedure (Neumeister, Schulz, & Glodecki, 2017).

Different bone quantity caused by vertical and horizontal bone atrophy can be simulated in the study approach as well as situations with low bone quality. According to students' evaluation the model showed a good approach in the design of the cortical and cancellous bone. In particular this is an advantage compared to other implantology models were a monobloc was used as bone fragment (Güth et al., 2010; Nicot et al., 2019).

The cortical bone was evaluated comparable in hardness by drilling. However, the ability to compress the cancellous bone by implant insertion was missing. To simulate cancellous bone even more realistically, a printed insert made of a more flexible material with a fine honeycomb structure might be suitable. Using a clinical CBCT as a basis, the practitioner is able to simulate the complete digital workflow from CAD planning of the implant positioning down to the implant drilling by different surgical guides on the same model compared to a real guided implantology (Cassetta, Giansanti, Di Mambro, Calasso, & Barbato, 2013).

The fact that the model is based on radiological examination of real patients enables students to understand and manage the problems of different anatomical complexities such as bone contour, atrophy, and practice more often before the first real implantation.

In total, the model gave added value to the curriculum. However, some limitations should be mentioned. Implantology on a real patient is affected by soft hard tissue interferences, saliva, blood, and patient movement which cannot be simulated yet that easily. Also, the printed resin also did not have the same physical properties as bone and tooth substance, which can lead to clinical changes in the implant bed drilling, and insertion of the implant as well as seat of the guides.

The workflow for creating the model was feasible by using open‐source software solutions that were user‐friendly and free of charge. The specific 3D printer can be used in the dental laboratory for a wide range of possible applications. Taking into account the results of this investigation, one has to state that the presented workflow enables dental schools to design and manufacture customized training models that offer highly realistic simulations. Nevertheless, there are still issues to be solved, such as hardness and radiopacity, that cannot overcome the benefit of extracted human teeth or cadaver models yet.

## 5 CONCLUSIONS

3D printing technology offers new possibilities to dental schools by creating their own customized teaching models according to the specific curricula. The presented workflow is a feasible way of using DICOM‐data as a basis for such models. The presented model which enables several interdisciplinary treatment scenarios, was rated to be realistic and give the patient a more central place in the education.

## CONFLICT OF INTEREST

The authors state to have no conflict of interest.

## APPENDIX A RESULTS OF SURVEY ON ENDODONTIC TREATMENT (48 PARTICIPANTS, NUMBER IN BRACKETS INDICATED NUMBER OF RESPONSES)

1. The design of the model is
  - highly realistic (14)
  - realistic (33)
  - not all realistic (1)
2. Radiograph diagnosis can be simulated
  - highly realistic (11)
  - realistic (35)
  - not all realistic (2)
3. How similar is the radiopacity of the training teeth in comparison to real teeth?
  - 75–100% (highly realistic) (28)
  - 50–74% (realistic) (14)
  - <50% (not at all realistic) (6)
4. The single steps of working length determination by using an electronic apex locator can be simulated
  - highly realistic (22)
  - realistic (25)
  - not all realistic (1)
5. The single steps of root canal instrumentation can be simulated
  - highly realistic (16)
  - realistic (29)
  - not all realistic (3)
6. How similar is the hardness of the training tooth in comparison to real teeth?
  - 75–100% (highly realistic) (11)
  - 50–74% (realistic) (29)
  - <50% (not at all realistic) (8)
7. Does the training bring an added value to the curriculum?
  - a high value (25)
  - some value (22)
  - no value at all (1)
8. How well do you feel prepared for endodontic treatment after the training in comparison to before?
  - better (38)
  - the same (10)
  - worse (0)

## APPENDIX B RESULTS OF SURVEY ON POST INSERTION (34 PARTICIPANTS, NUMBER IN BRACKETS INDICATED NUMBER OF RESPONSES)

9 The design of the model is
  - highly realistic (8)
  - realistic (24)
  - not all realistic (2)
10 Radiograph diagnosis can be simulated
  - highly realistic (14)
  - realistic (18)
  - not all realistic (2)
11 How similar is the radiopacity of the training tooth in comparison to real teeth?
  - 75–100% (highly realistic) (13)
  - 50–74% (realistic) (19)
  - <50% (not at all realistic) (2)
12 The single steps of post insertion can be simulated
  - highly realistic (15)
  - realistic (14)
  - not all realistic (5)
13 How similar is the hardness of the training tooth in comparison to real teeth?
  - 75–100% (highly realistic) (4)
  - 50–74% (realistic) (18)
  - <50% (not at all realistic) (12)
14 Does the training bring an added value to the curriculum?
  - a high value (7)
  - some value (26)
  - no value at all (1)
15 How well do you feel prepared for post insertion after the training in comparison to before?
  - better (26)
  - the same (8)
  - worse (0)

## APPENDIX C RESULTS OF SURVEY ON IMPLANT DENTISTRY (12 PARTICIPANTS, NUMBER IN BRACKETS INDICATED NUMBER OF RESPONSES)

16 The design of the model is
  - highly realistic (0)
  - realistic (12)
  - not all realistic (0)
17 By training with the model, the single steps of implant dentistry became more understandable to me
  - strongly agree (9)
  - partially agree (3)
  - agree not at all (0)
18 The single steps of dental implantology can be simulated
  - highly realistic (1)
  - realistic (8)
  - not all realistic (3)
19 How similar is the distinction between cortical and compact bone in comparison to real bone?
  - highly similar (0)
  - similar (8)
  - not at all similar (4)
20 Does the training bring an added value to the curriculum?
  - a high value (5)
  - some value (7)
  - no value at all (0)
21 Was the surgical guide helpful during implant insertion?
  - very helpful (12)
  - fairly helpful (0)
  - not at all helpful (0)
22 How well do you feel prepared for implant dentistry after the training in comparison to before?
  - better (4)
  - the same (8)
  - worse (0)
